# Dual‐specificity phosphatase 8: A gatekeeper in hypothalamic control of glucose metabolism in males

**DOI:** 10.1111/jdi.13561

**Published:** 2021-05-26

**Authors:** Keizo Kaneko, Hideki Katagiri

**Affiliations:** ^1^ Department of Metabolism and Diabetes Tohoku University Graduate School of Medicine Sendai Japan

Type 2 diabetes is a multifactorial chronic disease triggered by genetic and/or environmental factors. Insulin resistance in peripheral organs is one of the major causes of type 2 diabetes. The brain organizes whole‐body metabolism, connecting tightly with the periphery^1^. The hypothalamus is a key brain center that controls autonomic nerve balance and the hormonal system, including the hypothalamic–pituitary–adrenal (HPA) axis, by modulating hepatic glucose production, pancreatic insulin secretion and peripheral insulin sensitivity. Hypothalamic neuronal subpopulations within the arcuate nucleus and paraventricular nucleus are major targets for diabetes and obesity research. For example, pro‐opiomelanocortin and agouti‐related peptide neurons in the arcuate nucleus, both of which have dense connection with corticotrophin‐releasing hormone (CRH) neurons in the paraventricular nucleus, participate in the HPA axis and autonomic nerve activity. Insulin is an adiposity signal that exerts modulatory functions involving pro‐opiomelanocortin and agouti‐related peptide neurons. Nasal insulin administration stimulates hypothalamic insulin signaling to suppress hepatic glucose production and enhance insulin sensitivity in lean individuals, while having little effect in the obese, indicating hypothalamic insulin resistance to be the etiology of metabolic disorders. It is thus an important issue in diabetes research to clarify the mechanism(s) underpinning the hypothalamic role in systemic metabolism under insulin resistant conditions.

Dual‐specificity phosphatase 8 (DUSP8) is one of the mitogen‐activated protein kinase (MAPK) phosphatases, controlling MAPK activity of c‐Jun N‐terminal kinase (JNK), p38 and extracellular signal‐regulated kinase. A large‐scale genome‐wide association study and Metabochip meta‐analysis showed minor allele carriers of the *DUSP8* single‐nucleotide polymorphism rs2334499 to be moderately associated with the risk of type 2 diabetes only in men^2^. *Dusp8* is reported to be highly expressed in the adult human brain, but whether or how DUSP8 participates in diabetes development has not been clarified.

Recently, Schirever *et al*.^3^ tackled the challenge of clarifying the role of hypothalamic DUSP8 in systemic glucose metabolism and hypothalamic insulin sensitivity in mice and humans. First, the authors confirmed the brain to be a *Dusp8*‐abundant organ, and found that the *Dusp8* level in the hypothalamus was upregulated by body adiposity in mice. Consistently, *Dusp8* in the infundibular nuclei of type 2 diabetes patients was shown to be significantly increased, as compared with non‐diabetic control individuals in the Netherlands Brain Bank. The authors then explored the involvement of DUSP8 in systemic glucose or energy metabolism using mice with global deletion of *Dusp8* (*Dusp8*‐knockout [KO] mice). *Dusp8*‐KO mice fed a standard diet did not differ in metabolic phenotypes from the wild‐type control mice. In contrast, under high‐fat diet (HFD) feeding conditions, glucose tolerance and insulin resistance were exacerbated with no alteration of either insulin secretory capacity or body weight in male, but not in female, *Dusp8*‐KO mice. Next, *Dusp8* overexpression in the mediobasal hypothalamus, using an adeno‐associated virus vector, reversed the HFD‐induced insulin resistance in *Dusp8*‐KO mice. These findings highlighted the role of hypothalamic DUSP8 in glucose metabolism in obesogenic environments, although these DUSP8 effects were observed selectively in male mice.

To identify the neuronal subpopulations responsible for the DUSP8 effects, the authors used mice with conditional ablation of *Dusp8* in CRH‐producing neurons (*Dusp8^CRH‐Cre^* KO mice). Glucose tolerance of HFD‐fed *Dusp8^CRH‐Cre^* KO mice was impaired as compared with wild‐type controls, but the extent of the impairment was milder than that of global *Dusp8*‐KO mice, suggesting other hypothalamic neuronal subpopulations to also be involved, although conditional *Dusp8* deletion in agouti‐related peptide or pro‐opiomelanocortin neurons produced no distinct metabolic phenotypes.

The authors additionally explored the mechanism linking the hypothalamus with metabolic phenotypes at the whole‐body level. The genetic components of glucocorticoid signaling were upregulated in the pituitary, hypothalamus, liver and muscle. Both dexamethasone suppression and CRH stimulation tests showed HPA hyper reactivity in HFD‐fed *Dusp8*‐KO mice. Chemical adrenalectomy by metyrapone treatment reversed the impaired glucose tolerance in HFD‐fed *Dusp8*‐KO mice. Furthermore, the authors showed inhibitory effects of DUSP8 on the phosphorylation of Ser226 of the glucocorticoid receptor (GR^ser226^) that suppresses GR activity. Anisomycin (general MAPK activator and endoplasmic reticulum stressor)‐induced enhancement of GR^ser226^ phosphorylation was prevented by *Dusp8* overexpression *in vitro*. The role of DUSP8 in GR activity was also confirmed by the GR luciferase reporter assay. In addition, GR^ser226^ phosphorylation in the hypothalamus was elevated in HFD‐fed *Dusp8*‐KO mice. These results suggested the compromised GR‐mediated negative feedback of the HPA axis to be related to dysregulation of glucose homeostasis induced by *Dusp8* deletion.

As JNK, among the three MAPKs (JNK, p38 and extracellular signal‐regulated kinase), was shown to be the prominent downstream target of hypothalamic DUSP8 signaling toward GR^ser226^ phosphorylation, the authors studied the involvement of JNK by using mice with codeletion of *Jnk1* and *Dusp8* (*Jnk1*‐*Dusp8*‐dKO), and found that the exacerbation of HFD‐induced glucose intolerance and hypercorticosteronemia observed in HFD‐fed *Dusp8*‐KO mice were abrogated by additional deletion of *Jnk1*, suggesting that JNK mediates DUSP8 effects on systemic metabolism.

Interestingly, augmentation of hypothalamic Akt phosphorylation after an acute insulin challenge was blunted in HFD‐fed *Dusp8*‐KO males, as compared with HFD‐fed wild‐type controls. The evaluation of hypothalamic insulin sensitivity measured by cerebral blood flow based on magnetic resonance imaging after nasal insulin administration in 47 non‐diabetic volunteers showed a significant association between insulin‐dependent cerebral blood flow suppression and *DUSP8* polymorphism, whereas the association was observed only in male subjects. These findings indicate that DUSP8 is involved in hypothalamic insulin sensitization in males.

Based on these results, the authors hypothesized that DUSP8‐JNK signaling in the hypothalamus, including CRH‐producing neurons, affects HPA axis reactivity involving the GR phosphorylation level, thereby modulating systemic glucose tolerance in male mice (Figure 1). Thus, this line of research provides a novel insight into the role of the hypothalamus in systemic glucose metabolism. In addition, HFD‐fed *Dusp8*‐KO mice showed decreased norepinephrine turnover rates in the liver, muscle and pancreas. There are, however, further questions that are necessary to be answered before the underpinnings of this process are fully understood.

**Figure 1:**
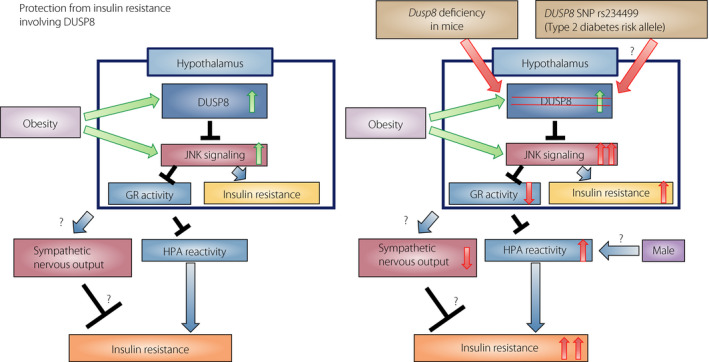
Proposed mechanism of the involvement of hypothalamic dual‐specificity phosphatase 8 (DUSP8) c‐Jun N‐terminal kinase (JNK) signaling in glucose metabolism under conditions of obesity. Under conditions of obesity, both *Dusp8* expression and JNK are upregulated in the hypothalamus. The elevation of *Dusp8* in obesity might be a protective response against further activation of JNK signaling to maintain hypothalamic–pituitary–adrenal (HPA) axis integrity, thereby preventing exacerbation of type 2 diabetes (left). DUSP8 deficiency and polymorphism might impair the preventive mechanism, leading to further insulin resistance (right). GR, glucocorticoid receptor; SNP, single‐nucleotide polymorphism.

First, it is still unclear how *DUSP8* polymorphism affects the role of hypothalamic DUSP8‐JNK signaling in glucose metabolism, leading to diabetes development. DUSP8 was originally identified as an immediate early gene, and its expression could be regulated by gene transcription and protein stability^4^. In addition to the expression level, the degree of DUSP8 phosphatase activity in obesogenic environments should also be considered. In fact, it is not clear whether *DUSP8* polymorphism affects its expression level and/or phosphatase activity. Given that both *Dusp8* expression and JNK phosphorylation in the hypothalamus are reportedly upregulated in obesity, hypothalamic *Dusp8* expression might be involved in a compensatory response to maintain HPA axis integrity, thereby preventing further exacerbation of insulin resistance. This mechanism might not fully function in individuals with type 2 diabetes risk allele of the *DUSP8* gene. Further research is required to untangle the complex roles of proposed hypothalamic DUSP8‐JNK signaling in glucose homeostasis and, ultimately, thereby clarify the mechanism linking *DUSP8* polymorphism with development of type 2 diabetes in humans.

The next question is how DUSP8 is involved in the brain‐orchestrated neural inter‐organ communication governing systemic glucose metabolism. Interestingly, besides the HPA axis feedback alteration, the authors showed decreased sympathetic tones in multiple metabolic organs in HFD‐fed *Dusp8*‐KO mice. The sympathetic nervous system is reportedly related to liver glucose production, muscle glucose uptake and pancreatic insulin secretion. Furthermore, parasympathetic nerves also reportedly innervate important metabolic organs. The functions of and balance between these two efferent autonomic nervous systems, together with the afferent nerves, have key roles in transmitting metabolic information. At present, many researchers are vigorously exploring how these systems are involved in the obesity‐related metabolic disorders^1^. Intensive research will reveal the roles of DUSP8 in inter‐organ neural communication.

Finally, why the effects of DUSP8 on insulin sensitivity in both humans and mice are present only in males remains to be elucidated. Although the authors speculated that sex hormones are involved in the DUSP8‐mediated HPA axis reactivity, it seems unlikely that this mechanism explains the clear sex difference. Further intensive studies are required to explore this important issue. In addition, although a meta‐analysis showed that the association of *DUSP8* single‐nucleotide polymorphism re2334499 with type 2 diabetes was limited to males^2^, a genome‐wide association study, initially identifying *DUSP8* as a type 2 diabetes risk gene, showed that the type 2 diabetes risk associated with re2334499 was inherited with the paternal allele^5^. Thus, details of the complexity of the association between type 2 diabetes susceptibility and *DUSP8* polymorphism await future research.

In summary, *DUSP8*, a type 2 diabetes risk gene, might be a novel gatekeeper that functions against deleterious metabolic effects induced by JNK activation in obesity. Further research is expected to clarify the physiological significance of hypothalamic DUSP8‐JNK signaling in complex brain‐orchestrated inter‐organ communication necessary for systemic metabolism.

## Disclosure

The authors declare no conflict of interest.
